# Contributions of rational soil tillage to compaction stress in main peanut producing areas of China

**DOI:** 10.1038/srep38629

**Published:** 2016-12-09

**Authors:** Pu Shen, Zhengfeng Wu, Chunxiao Wang, Sheng Luo, Yongmei Zheng, Tianyi Yu, Xuewu Sun, Xiushan Sun, Caibin Wang, Xinhua He

**Affiliations:** 1 Shandong Peanut Research Institute, Qingdao 266100, China; 2 Yantai Academy of Agricultural Sciences, Yantai 264000, China; 3 Southwest University, Chongqing 400715, China

## Abstract

Tillage intensities largely affect soil compaction dynamics in agro-ecosystems. However, the contribution of tillage intensities on compaction changes in underground peanut (*Arachis hypogaea*) fields has not been quantified. We thus aimed to better understand the role of soil tillage intensities in mitigation of compaction stress for peanuts. Using three field tillage experiments in major Chinese peanut producing areas, we quantified the effects of (1) no tillage, (2) shallow (20 cm) plowing, (3) deep (30 cm) plowing and (4) deep (30 cm) loosening on changes in soil bulk density at 0–10 cm, 10–20 cm and 20–30 cm depths, roots and pods growth, and nutrient accumulation. Results showed that tillage management effectively mitigated soil compaction stress for peanut growth and production. Greater beneficial improvement for the underground growth of roots and pods, and N accumulation ranked as deep plowing > shallow plowing and deep loosening. Respective increases of 7.5% and 4.6% in root biomass productions and peanut yields were obtained when soil bulk density was decreased by 0.1 g cm^−3^. Our results suggest that the mitigation of soil compaction stress by deep plowing could be a key tillage strategy for increasing peanut yields in the field.

Soil compaction, one of the greatest challenges for crop production over the world, severely inhibits crop growth and thus decreases crop productivity^1^,^2^,^3^. With the occurrence of universal mechanical rolling, high cropping indexes and irrational usage of chemical fertilizers and water, global farmlands are facing serious problems of compaction stresses in agricultural ecosystems^4^,^5^,^6^,^7^,^8^. Thus, the compaction mitigation by field management is recognized as an effective strategy^9^,^10^,^11^. Soil compaction dynamics mainly depend on the degrees and patterns of soil disturbance. While soil disturbance always results in a decrease in compaction, the relationship between these two parameters is not straightforward^12^. For example, some disturbances could decrease top soil compaction, while others were even able to remove the compaction in deep soil. As a general rule, a higher tillage intensity to a deeper soil profile could significantly affect soil properties and hence plant growth, compared to lower tillage intensity or no tillage practice^13^,^14^,^15^.

The growth and distribution of roots were largely affected by soil compaction intensities, which enhanced the root resistance and deteriorated the soil physical characteristics (e.g., soil aeration and moisture content)^16^,^17^,^18^,^19^. Moreover, an arrested development of roots had substantial effects on the nutrient uptake and organic composition of crops^20^,^21^,^22^,^23^. Except for root disruption, other underground parts were directly inhibited by soil compaction for underground crops^24^,^25^. The peanut, one of the most important underground crops (annual yields of 40 million tones globally and 50% of these are from China), also faces such a soil compaction issue, which has brought potential risks for edible oil security in China and worldwide^9^,^26^.

Recent studies have showed the important role of soil tillage in solving the compaction problem^10^,^15^. This could be attributed to the breakup of soil compaction, improvement of soil aeration and moisture, and activation of soil nutrients^27^,^28^. However, there has been limited quantification of appropriate tillage management strategies to mitigate soil compaction. For example, which tillage system or intensity is beneficial to coordinate the growth of the roots and pods of peanuts, and simultaneously improve nutrient accumulation and yield?

In this study, we reported on a comprehensive and quantitative analysis of data generated from three soil tillage treatments in the main peanut producing areas of China. We analyzed soil and plant samples from three field sites and determined the soil bulk density, root distribution, nutrient accumulation and peanut yield. The objective of this study was to address different roles of three tillage intensities (NT: no tillage; SP: shallow plowing; DP deep plowing; DL: deep loosening) in mitigating soil compaction stress and then to relate soil compaction mitigation to root growth and peanut yield. The expected results could promote a better understanding of tillage management strategies to increase belowground crop yields under increasing soil compaction scenarios with large scale machinery tillage activities.

## Results

### Soil compaction

**Figure 1:**
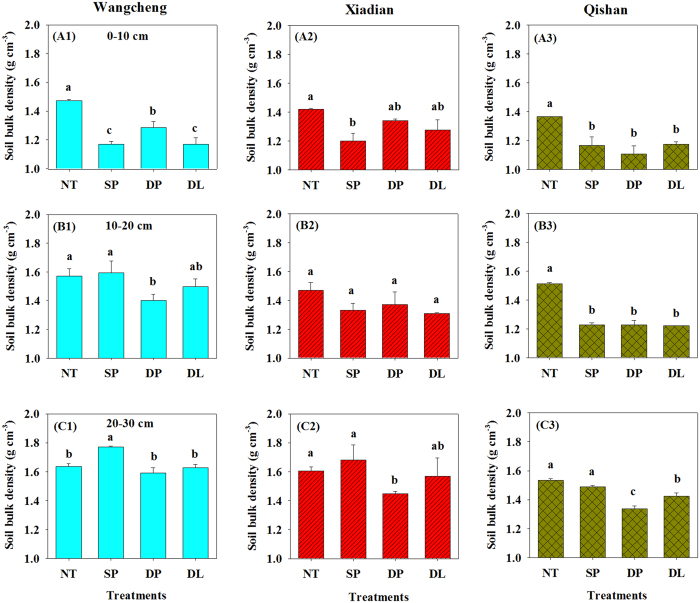
Soil bulk densities with different soil layers (A1–A3: 0–10 cm, B1–B3: 10–20 cm, C1–C3: 20–30 cm) at three peanut field sites. NT: no tillage; SP: shallow plowing; DP deep plowing; DL: deep loosening. Different letters above the bars indicate significant differences (*P* < 0.05) among treatments.

Soil bulk density at the three sites at a depth of 0–10 cm was significantly higher under no tillage than under tillage intensities (Fig. 1). For example, the average values of soil bulk density under SP, DP and DL were decreased by 17.9%, 10.4% and 15.9%, respectively, compared with the NT treatment at Wangcheng, Xiadian and Qishan, respectively. For soil at a depth of 10–20 cm, the bulk density was 6.6–18.8% lower under DP than under NT at all three sites, though without significant differences at Xiadian. In contrast, soil bulk density was 4.8–8.3% higher under SP than under NT at Wangcheng and Xiadian. Soil bulk density under DP was lowest, ranging from 1.34 to 1.59 g cm^−3^ at a depth of 20–30 cm among the four treatments at the three sites.

### Root distribution and morphological characteristics

**Figure 2:**
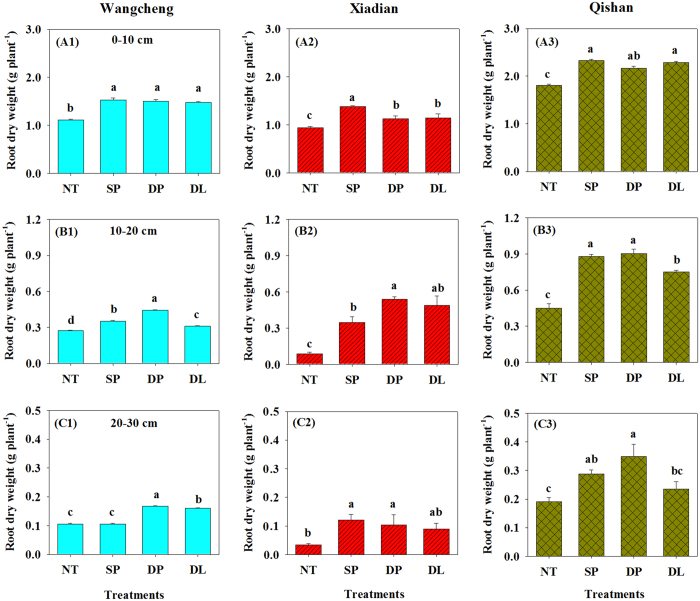
Root dry weights within different soil layers (A1–A3: 0–10 cm, B1–B3: 10–20 cm, C1–C3: 20–30 cm) at the three experimental sites. NT: no tillage; SP: shallow plowing; DP deep plowing; DL: deep loosening. Different letters above bars indicate significant differences (*P* < 0.05) among treatments for the same field site.

Root dry weight at 0–10 cm depth was the lowest (1.1 g plant^−1^) under NT, while it was similar between the three tillage treatments (~1.5 g plant^−1^) (Fig. 2). Root weights were higher at 10–20 cm and 20–30 cm depth under DP than under NT, SP and DL at Wangcheng. The root weights accounted for 74.7%, 18.2% and 7.1% at 0–10 cm, 10–20 cm and 20–30 cm depths, respectively.

Root weights at 0–30 cm in Xiadian were the lowest under NT. Root weights at 0–10 cm were higher under SP than under DP, while they were highest under DP at both 10–20 cm and 20–30 cm. Higher proportions of root biomass production at 10–20 cm were observed under DP (30.4%) and DL (28.4%) than under SP (18.8%) and NT (8.1%), although most of the roots (0.9–1.4 g plant^−1^) were distributed at a 0–10 cm depth.

Root growth was also decreased under NT at Qishan. The root weights were always highest under SP and DP and accounted for 63.4–66.6%, 25.2–26.4% and 8.2–10.2% at 0–10 cm, 10–20 cm and 20–30 cm, respectively. A lower root biomass was observed under DL than under DP at both 10–20 cm and 20–30 cm depths.

**Figure 3:**
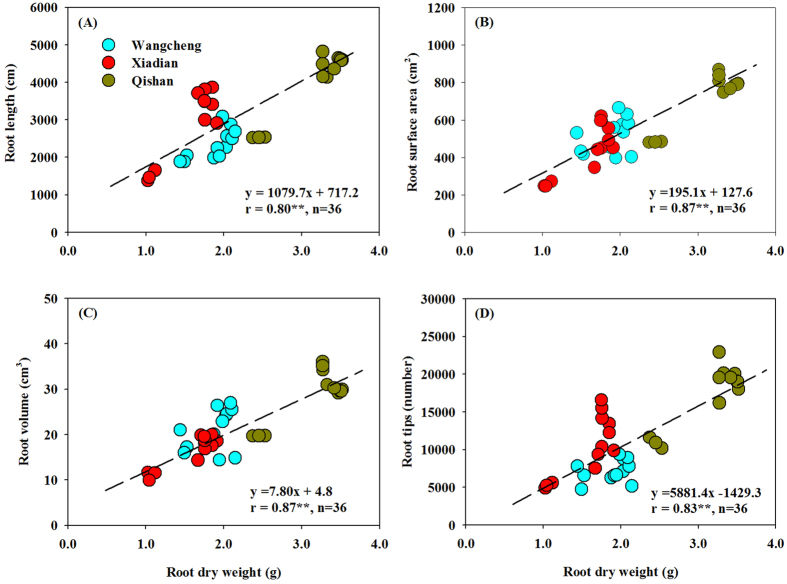
Linear correlations between root dry weight (0–30 cm soil depth) and root length, surface area or volume and tips. Values are Pearson correlation coefficients. Significant correlations are marked with two asterisks (*P* < 0.01).

Correlation analyses indicated that root morphological characteristics were significantly affected by root weights (Fig. 3). Root length, surface area, volume and tips were positively correlated with root weights at 0–30 cm depth (*P *< 0.01). With an increase of 1.0 g in root weight, the number of root tips could be increased by 5,881, while root length, surface area and volume could be increased by 1,080 cm, 195 cm^2^ and 7.8 cm^3^, respectively. Obviously, root morphological characteristics (length, surface area, volume and tips) were lower under NT than under DP, SP and DL at the three sites.

### Pod growth traits

Four pod traits, including pod length, width, hundred pod weights, and pod numbers, varied largely under different tillage treatments (see Fig. S1). Significantly higher growth traits generally followed the order as DP > SP and DL > NT (Table 1). For instance, compared with the other tillage treatments, pod size was the smallest, while pod length and width were generally decreased by 8.9–11.9% under NT at these three sites. Compared with NT, the weight of one hundred pods was the highest under DP and increased by 44.7%, 35.8% and 13.2% at Wangcheng, Xiadian and Qishan, respectively. The pod numbers were higher under DP at Qishan, while they were similar among tillage treatments at Wangcheng and Xiadian. In addition, the kernel rate was also similar under NT to other tillage treatments at three sites.

### N, P, K accumulations by plant

**Figure 4:**
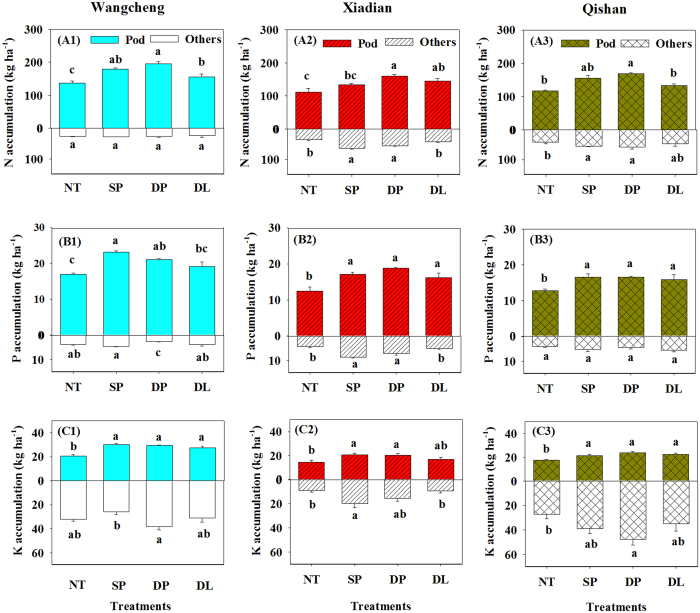
Nitrogen (N), phosphorus (P) and potassium (K) accumulations by peanut pods and other parts of peanut at three field sites. NT: no tillage; SP: shallow plowing; DP deep plowing; DL: deep loosening. Different letters above or under the bars indicate significant differences (*P* < 0.05) among treatments.

Our data exhibited a profound positive relationship between nutrient accumulation and tillage treatments. Total N accumulation by pod and other tissues of the peanut were significantly higher under tillage treatments (180–228 kg ha^−1^) than under no tillage (146–163 kg ha^−1^) at the three sites (Fig. 4). The N accumulation in pod under DP was the highest, which increased by 44.1%, 21.2% and 11.9% than those under NT, DL and SP, respectively. Compared with the no tillage control, tillage treatments also increased tissue P and K accumulations (Fig. 4). For instance, P and K accumulations in pods were 24.7% to 41.6% higher under SP, DP and DL than under NT. However, pod P and K accumulations were similar among the three tillage treatments.

### Peanut yields

**Figure 5:**
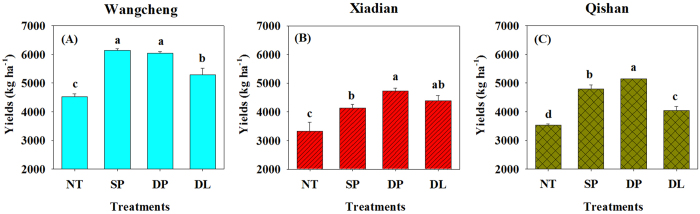
Yields of peanut at three field sites. NT: no tillage; SP: shallow plowing; DP deep plowing; DL: deep loosening. Different letters above the bars indicate significant differences (*P* < 0.05) among treatments.

Yields for the three experimental sites were presented in Fig. 5. Compared with the three tillage treatments, the lowest peanut yields were under NT at all three sites. For instance, yields were 3,326–3,533 kg ha^−1^ under NT at Xiadian and Qishan but 4,130–5,142 kg ha^−1^ under the other three tillage treatments. At Wangcheng, yields under DP, SP and DL ranged from 5,284 to 6,142 kg ha^−1^, which were higher than 4,521 kg ha^−1^ under NT. Meanwhile, yields at the three sites were the highest under DP while they were similar between SP and DP at Wangcheng and between DP and DL at Xiadian.

## Discussion

Soil compaction stress exerts dominant effects on the plant growth, especially underground, all over the world^5^,^29^,^30^. Peanut, one of the most important oil crops, is always inhibited by compaction stress^25^,^26^. Soil tillage, as a major agricultural management, can effectively improve the soil structure and mitigate the growth of plants in plant-soil systems under soil compaction stress. Based on the response of peanut plants to various tillage intensities, the generated result is required for rational field management by taking into account the soil depths of tillage intensities.

Our data showed that soil compaction stress for peanut emerged under no tillage (Figs 1,2,3,4 and 5, Table 1), which was consistent with the typical trends observed in wheat, maize, etc.^31^,^32^,^33^. However, soil compaction stress could be eliminated under only two rounds (2014 and 2015) of various tillage intensities followed by several decades of peanut cropping or by rotation with maize and/or wheat under traditional SP tillage. Hence these results could provide a general idea for rational peanut field management. There was evidence that soil became loose and bulk density declined with the use of mechanical plows in field management, which would lead to the rational soil structure and physicochemical properties^34^,^35^,^36^. In the present study, large differences were found in the effects of various tillage intensities on soil bulk density (Fig. 1). At the three experimental sites, DP treatment resulted in the lowest soil bulk density at 0–30 cm depth, whereas SP and DL treatments decreased soil bulk density only at 0–10 cm or 10–20 cm depths. This was obviously due to the plow depths of tillage intensities. In addition, a new plow hardpan had been formed under SP (Fig. 1), which could generate potential compaction risks at ~20 cm soil depth in the near future.

**Figure 6:**
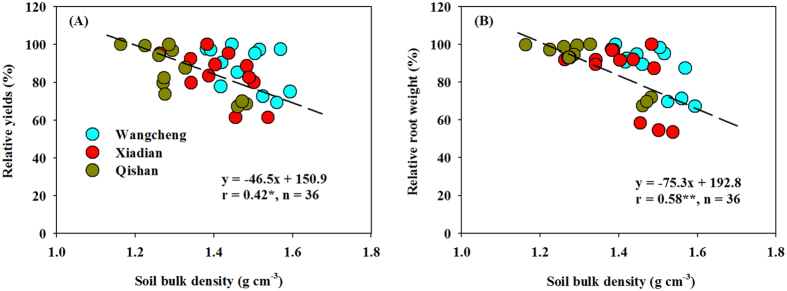
Linear correlations between averaged soil bulk density (0–30 cm depth) and relative yields and root biomass production (0–30 cm soil depth). Relative yields or root weight were obtained from the ratio of original values to maximum values. The data are Pearson correlation coefficients. Significant correlations are marked with one asterisk (*P* < 0.05) and two asterisks (*P* < 0.01).

There have been a number of root change studies from numerous crops under soil tillage intensities^19^,^32^,^37^,^38^. However, detailed analyses of the responses of root and other underground parts (e.g., pod) to soil compaction are limited. Our study demonstrated that soil tillage improved the growth of root and pod compared with NT treatment (Fig. 2, Table 1). Root dry weight and pod characteristics were always the highest under DP. These results were confirmed with the changes in soil bulk density, i.e., soil bulk density at 0–30 cm significantly negatively related with root biomass and yield of peanuts (Fig. 6). Given that soil bulk density was decreased by 0.1 g cm^−3^, peanut root biomass and yield would be increased by 7.5% and 4.6% (*P *< 0.05). These quantitative relationships further indicated the important role of tillage intensities, especially the deep plowing in peanut fields.

The accumulations of N, P and K were significantly lower under NT than under other three tillage treatments, with the highest accumulation under DP at the three sites (Fig. 4). This was mainly attributed to the decrease of soil bulk density and the accompanied improvement of aeration and moisture, which promoted root interception and nutrient uptake abilities by enlarging the root length, surface area, volume, and tip number (Fig. 3). On the other hand, the activities of nodule N_2_-fixing bacteria might be enhanced by better soil conditions under DP, and N availability to plant growth could then be improved^12^,^24^,^39^. However, there were almost no significant differences in P and K accumulations in pods among these three tillage intensities. For some mechanisms, the peanut pod itself could have the ability to absorb more nutrients and mitigate compaction stress at 0–10 cm soil depth under SP and DL (Fig. 4).

The DP tillage improved the physical structure at 0–30 cm depth, which resulted in a higher peanut yield through an enhanced root and pod growth and nutrient uptake, although the operation of the deep plowing cost more due to energy consumption. However, tillage activities could also generate potential compaction risks in the plow layer (either 20 cm or 30 cm in this study, see Fig. 1-B1, C2 and C3) in the long term. As a result, maintaining a critical point of soil compaction stress could be one of the key future peanut field management strategies.

## Materials and Methods

### Experimental locations, climate and soil properties

The three tillage experimental sites at Wangcheng (N 36°48′, E120°29′), Xiadian (N 37°13′, E120°25′), and Qishan (N 37°15′, E120°22′) are located in the major peanut producing area of Shandong, China. This area has a mean annual temperature and precipitation ranging from 11.5 °C to 11.7 °C and from 635.8 mm to 671.1 mm (mostly between May and September), respectively. The soil is a typical brown soil (Haplic Luvisol, FAO Soil Taxonomic System) that has been developed from the same parent material (i.e., an acid rock)^40^. This soil at the three sites had a range of pH from 4.8 to 5.8 and bulk densities from 1.49 to 1.54 g cm^−3^. Other physicochemical properties varied among these sites (Table 2). The major cropping system has been a rotation of peanut with wheat and maize for several decades before this experiment.

### Experimental design

In a split field experiment design with three replicates for each tillage treatment or plot (110–130 m^2^), four tillage treatments, including the traditional 20 cm shallow plowing (several decades) at each of the three sites, had been established since 2014: (1) no tillage (NT), (2) shallow plowing at a 20 cm depth (SP), (3) deep plowing at a 30 cm depth (DP), and (4) deep loosening at a 30 cm depth (DL). The field tillage managements of SP and DP were performed by a plow machine (1LYF-435, Yucheng Dadi Machinery Co., Ltd., China), and the DL was by a subsoiler machine (1SL-300, Shandong Dahua Machinery Co., Ltd., China). The tillage intensities were measured before winter in November 2014. On May 12–14, 2015 two seeds of peanut (*Arachis hypogae*a ‘Huayu 33’) were sown inside one seed hole on the peanut ridge (85 cm width × 8.5 cm depth), and the distance between two holes were 20 cm. The fertilization and other field managements were consistent among these four tillage treatments. According to the local farming practice, the fertilization rates were 112.5 kg N ha^−1^, 49.1 kg P ha^−1^ and 123.7 kg K ha^−1^, and the fertilizers applied were 750 kg tri-elements chemical fertilizer (15.0% N, 6.5% P and 12.5% K) and 60 kg potassium sulfate (50.0% K and 17.5% S) per hectare.

### Plant and soil sampling and analyses

Soil and plant sampling were conducted on peanut harvest days (September 23–25, 2015). An area of 400 cm (length) × 85 cm (width) was sampled for determination of peanut yield and pod characteristics. Typically, four plants were randomly selected to determine nutrient contents of tissues (seeds and other plant parts). Plant tissues were dried (70 °C), ground (<0.15 mm), and digested with H_2_SO_4_-H_2_O_2_ for the determination of N, P and K^41^. Soil bulk density was determined by the cutting ring method at 0–10 cm, 10–20 cm and 20–30 cm depths. Root samples were also collected at 0–10 cm, 10–20 cm and 20–30 cm depths during the maximum root growth period (12–14 August, 2015). Root length, surface area, volume and tip number on fresh roots were determined by an Optical Scanner STD 4800 (Epson, Japan) and WinRHIZO^®^ Regular 2009 (Regent Instruments Inc., Canada). The root biomass was recorded after being oven-dried to a consistent weight.

### Calculation and statistical analyses

Relative yield or root weight at each site was calculated according to Bai *et al*.^42^, where data were obtained from the ratio of original values to maximum values. Differences in soil bulk density, root weight, pod characteristics, nutrient content and yield among treatments were subjected to analyses of variance (ANOVA) by using SAS 8.0 (SAS, Inc., Cary NC). The least significant difference (LSD) test was used to separate the differences between treatments at *P* < 0.05. The significances of correlations across root weight and root morphological characteristics, soil bulk density and root weight or yield were shown at *P* < 0.05 or 0.01.

## Additional Information

**How to cite this article**: Shen, P. *et al*. Contributions of rational soil tillage to compaction stress in main peanut producing areas of China. *Sci. Rep.* **6**, 38629; doi: 10.1038/srep38629 (2016).

**Publisher's note:** Springer Nature remains neutral with regard to jurisdictional claims in published maps and institutional affiliations.

## Supplementary Material

Supplementary Figure S1

## Figures and Tables

**Table 1:** Effects of soil tillage measurements on pod growth traits of peanut.

Sites	Treatments	Pod length.(cm)	Pod width.(cm)	Hundred pod weight (g)	Pod number (plant^−1^)	Kernel rate (%)
Wangcheng	No tillage	3.7 ± 0.1b	1.5 ± 0b	165.6 ± 5.7c	20.4 ± 0.4a	71.6 ± 1.3ab
	Shallow plowing	4.3 ± 0.1a	1.7 ± 0a	246.8 ± 4.8a	18.8 ± 0.2a	70.0 ± 0.5b
	Deep plowing	4.4 ± 0.1a	1.7 ± 0a	239.6 ± 4.2a	18.3 ± 1.0a	73.5 ± 0.3a
	Deep loosening	4.3 ± 0.1a	1.7 ± 0a	215.9 ± 5.9b	18.5 ± 1.3a	72.7 ± 0.6a
					
Xiadian	No tillage	3.8 ± 0.1b	1.5 ± 0c	153.3 ± 0.9c	16.1 ± 1.5a	69.8 ± 0.6ab
	Shallow plowing	4.3 ± 0.1a	1.7 ± 0b	179.0 ± 7.6b	17.3 ± 0.5a	67.2 ± 0.6b
	Deep plowing	4.2 ± 0.1a	1.8 ± 0a	208.3 ± 3.6a	17.0 ± 0.4a	70.0 ± 0.3a
	Deep loosening	4.1 ± 0.1a	1.7 ± 0b	184.8 ± 4.3b	17.7 ± 0.3a	68.8 ± 1.0ab
					
Qishan	No tillage	4.0 ± 0.1b	1.6 ± 0a	167.3 ± 6.3b	17.0 ± 1.1bc	61.7 ± 1.3a
	Shallow plowing	4.3 ± 0.1a	1.7 ± 0a	191.4 ± 2.5a	18.8 ± 0.7ab	64.9 ± 1.7a
	Deep plowing	4.4 ± 0.1a	1.7 ± 0a	189.4 ± 0.6a	19.5 ± 0.7a	64.6 ± 0.8a
	Deep loosening	4.3 ± 0.1a	1.7 ± 0a	185.1 ± 4.1a	16.3 ± 0.2c	61.0 ± 2.0a

Different letters indicate significant differences (*P* < 0.05) among treatments at the same field site.

**Table 2:** Basic parameters of geography, climate and chemical properties of soils (0–30 cm) at three peanut field sties.

Parameters	Wangcheng	Xiadian	Qishan
Geography and climate			
Latitude	N 36°48′	N 37°13′	N 37°15′
longitude	E 120°29′	E 120°25′	E 120°22′
Annual mean temperature (°C)	11.7	11.5	11.5
Annual mean precipitation (mm)	635.8	671.1	671.1
Soil properties before experiment in 2014			
Soil pH	5.8 ± 0.3	5.4 ± 0.4	4.8 ± 0.2
Soil bulk density (g cm^−3^)	1.54 ± 0.10	1.56 ± 0.13	1.49 ± 0.12
Alkaline hydrolysable N (mg kg^−1^)	58.5 ± 2.5	60.6 ± 3.2	73.7 ± 5.1
Available P (mg kg^−1^)	47.4 ± 2.9	32.4 ± 1.6	54.6 ± 2.4
Available K (mg kg^−1^)	66.1 ± 4.8	28.8 ± 1.4	51.9 ± 1.0
