# Supplementary material for: Contributions of rational soil tillage to compaction stress in main peanut producing areas of China
Source: Sci Rep. 2016 Dec 9;6:38629. doi: 10.1038/srep38629 (PMC5146654; doi:10.1038/srep38629)

Contributions of rational soil tillage to compaction stress in main peanut producing areas of China

Pu Shen[1], Zhengfeng Wu[1], Chunxiao Wang[2], Sheng Luo[1], Yongmei Zheng[1], Tianyi Yu[1], Xuewu Sun[1], Xiushan Sun[1], Caibin Wang[1]* & Xinhua He[3]*

[1]Shandong Peanut Research Institute, Qingdao 266100, China.

[2]Yantai Academy of Agricultural Sciences, Yantai 264000, China.

[3]Southwest University, Chongqing 400715, China.

Correspondence and requests for materials should be addressed to C.B.W. (caibinw@126.com) or X.H.H. (xinhua.he@uwa.edu.au and hexinhua@swu.edu.cn)

Figure S1. Variation in pod sizes and shapes of peanuts under different soil tillage intensities at three field sites.

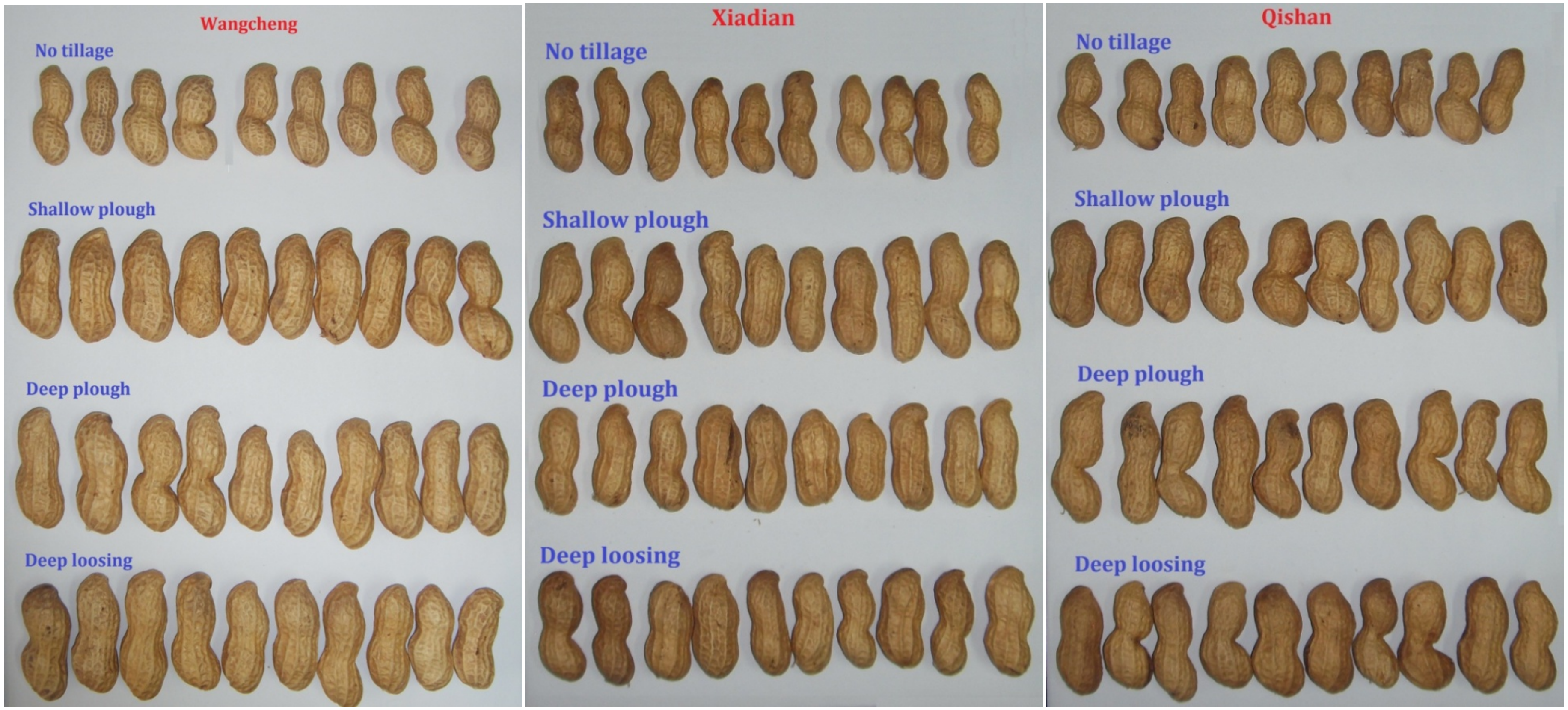

Supplement: Supplementary Figure S1 [file srep38629-s1.pdf]
